# Establishing a prediction model for lower extremity deep venous thrombosis in emergency inpatients in the post epidemic era

**DOI:** 10.3389/fsurg.2025.1543860

**Published:** 2025-04-07

**Authors:** Xiaodong Xia, Lei Hua, Yongqiang Zhang, Qing Tang, Jiaqi Xu, Shuxin Hua, Xiaohe Liu, Yanfen Chai, Lijun Wang

**Affiliations:** ^1^ Department of Emergency Medicine, Tianjin Medical University General Hospital, Tianjin, China; ^2^ Department of Craniocerebral Trauma and Critical Care Medicine, Tianjin Huanhu Hospital, Tianjin, China; ^3^ Department of Cardiology, Tianjin Medical University General Hospital, Tianjin, China

**Keywords:** emergency, post-epidemic era, lower extremity venous thrombosis, risk factor, prediction model

## Abstract

**Objective:**

This study aimed to analyze the risk factors of lower extremity deep vein thrombosis (LEDVT) in emergency inpatients in the post-epidemic era, and to establish a prediction model for identifying high-risk patients of LEDVT.

**Methods:**

Emergency inpatients admitted to our hospital from June 2022 to June 2023 were divided into two groups: the epidemic group and the post-epidemic group. The baseline characteristics, blood routine, liver and kidney function, blood coagulation function, and LE ultrasonography were compared between the two groups. Multivariate logistic analysis and receiver operating character (ROC) curve were used to establish and evaluate the effectiveness of a prediction model for LEDVT in the post-epidemic era.

**Results:**

A total of 967 patients were analyzed, including 388 cases in the epidemic group and 579 cases in the post-epidemic group. The portion of LEDVT cases in the post-epidemic group (33.2%) was significantly higher than that in the epidemic group (26.8%, *P* = 0.036). Binary Logistic regression analysis showed that age, smoking history, drinking history and glycosylated hemoglobin (HBA1c) were independent risk factors for thrombosis. The prediction model was established as *P* = 0.863 × age + 0.978 × smoking history + 0.702 × drinking history + 0.104 × HBA1c − 2.439. The area under the ROC curve was 0.718.

**Conclusion:**

The incidence of LEDVT in emergency inpatients in the post-epidemic era was significantly higher than that in the epidemic period. Age, smoking and drinking history, and glycosylated hemoglobin are at high risk for thrombosis.

## Introduction

Lower extremity deep vein thrombosis (LEDVT) is a serious medical condition in which blood clots occurs in the deep vein system of lower extremity (1). It is estimated that 67 per 100,000 general population suffers from DVT every year (2). LEDVT usually manifested as acute-onset pain, swelling and discomfort of lower extremity or gait disorder, and even lead to pulmonary embolism, disability mortality if left untreated (1, 3). Therefore, early diagnosis and treatment are crucial to minimize the adverse outcomes of LEDVT. Besides, differentiated treatment of those patients with low or high risk of developing LEDVT might avoid added costs and reduce the risk of anticoagulant therapy.

Combination of imaging tests and D-dimer tests were regarded as the first-line diagnosis method for DVT since 2012 (4). However, due to the ongoing SARS-CoV-2 infection pandemic worldwide, the incidence of thromboembolic events is increased with the elevated D-dimers and other bio humoral markers (5). Especially, nearly 30% of patients developed various long-term and persistent Post-Acute Sequelae of COVID-19, affecting daily life and work (6, 7). SARS-CoV-2 infection leads to increased release of inflammatory factors, microcirculation dysfunction, upregulation of hypoxic transcription factors, further results in coagulation dysfunction, venous thrombosis, pulmonary embolism and other serious complications (8–10). However, the comparison of the incidence of LEDVT in emergency inpatients and the influencing factors before and after the epidemic have not been reported.

In this study, the vascular ultrasound results of emergency inpatients during and after the SARS-CoV-2 epidemic were retrospectively reviewed, and the incidence of DVT at different periods and its influencing factors were preliminarily analyzed. The study might strengthen thrombosis screening in key populations and avoid the occurrence of malignant thrombotic events.

## Patients and methods

### Patients

The patients admitted to the Department of Emergency Medicine of Tianjin Medical University General Hospital for lower extremity venous vascular ultrasound From June 2022 to June 2023 were retrospectively reviewed. The inclusion criteria were patients aged >18 years old who completed lower extremity venous ultrasound examination at admission. The patients with >20% missing laboratory test results, multiple visits to emergency medicine wards, with previous venous thrombosis disease were excluded. The study was approved by the Ethics Committee of Tianjin Medical University General Hospital (ID: IRB2023-KY-129).

Following three years of the COVID-19 pandemic, the Comprehensive Group of the Joint Prevention and Control Mechanism under China's State Council released the Notice on Further Optimizing the Implementation of COVID-19 Prevention and Control Measures (commonly referred to as the “Ten New Guidelines”) on December 7, 2022 (11, 12). This policy marked a significant transition in China's pandemic response, signaling the shift from stringent containment measures to a post-epidemic phase characterized by normalized management. Based on this pivotal date, patients were categorized into two distinct groups: the epidemic group (pre-December 7, 2022) and the post-epidemic group (post-December 7, 2022).

### Data collection

The general information including age, sex, smoking history, drinking history, the laboratory tests including white blood cell count (WBC), red blood cell count (RBC), hemoglobin (HGB), fasting blood glucose (FBG), platelets (PLT), thrombin time (TT), prothrombin time (PT), international normalized ratio (PT-INR), activated partial thrombin time (APTT), D-Dimer, albumin (ALB), Globulin (GLO), alanine aminotransferase (ALT), aspartate aminotransferase (AST), lactate dehydrogenase (LDH), urea, creatinine (CREA), uric acid (URIC) and glycosylated hemoglobin (HbA1c) as well as lower extremity vascular ultrasound data, including whether there was thrombosis.

Smoking history includes current smokers (those who still smoke at the time of the study) and former smokers (those who have quit smoking for more than 6 months or 1 year), excluding never smokers (those who have smoked fewer than 100 cigarettes in their lifetime). Drinking history includes current drinkers (those who have consumed alcohol in the past year) and former drinkers (those who have quit drinking), excluding never drinkers (those who have consumed fewer than 12 units of alcohol in their lifetime, with one unit being approximately 10–12 g of pure alcohol).

Two independent members of the research group reviewed the data. If there was any inconsistency, the data were further reviewed by the research group leader to reach a consensus.

### Statistical analysis

Statistical analysis was executed in SPSS29.0. The measurement data following normal distribution were expressed as means ± standard deviation, and those following non-normal distribution were expressed as median and quartile. Comparisons between two groups were performed by independent-samples *t* test for normal distribution data and rank sum test for data with skew distribution. Comparisons of categorical data were tested by chi-square test. Single factor analysis, binary logistic regression and receiver operator characteristic curve analyses were used to establish prediction model. *P* < 0.05 indicated that the difference was statistically significant.

## Results

### Comparison of baseline characteristics

A total of 1003 patients performed lower extremity venous vascular ultrasound from June 2022 to June 2023. After excluding 36 patients, 967 patients were included in this study. The epidemic group included 388 patients (40.12%) and the post-epidemic group included 579 patients (59.88%).

The baseline characteristics of patients in the two groups is displayed in Table 1. The portions of patients older than 60 years old, with smoking history and drinking history were significantly higher in post-epidemic group than those in epidemic group (*P* < 0.05). No significant difference was observed in term of sex (*P* > 0.05).

**Table 1:** Baseline characteristics of patients in epidemic group and post-epidemic group.

Characteristics		Epidemic group *n* (%)	Post- epidemic group *N* (%)	*χ* ^2^	*P*
Age, years	<60	76 (19.6%)	72 (12.4%)	9.17	0.002
	≥60	312 (80.4%)	507 (87.6%)		
Sex	Male	191 (49.2%)	321 (55.4%)	3.60	0.058
	Female	197 (50.8%)	258 (44.6%)		
Smoking history	Yes	112 (28.9%)	211 (36.4%)	5.99	0.014
	No	276 (71.1%)	368 (63.6%)		
Drinking history	Yes	92 (23.7%)	185 (32%)	7.72	0.005
	No	296 (76.3%)	394 (68%)		

The blood routine, blood coagulation function, liver and kidney function and HBA1c also compared between the two groups. As shown in Table 2, the TT was significantly lower, while the PT, PT-INR, APTT and HbA1c were statistically higher in the post-epidemic group than those in the post-epidemic group (*P* < 0.05).

**Table 2:** Laboratory test results of emergency inpatients in epidemic group and post-epidemic group.

Characteristics	Epidemic group M (P25, P75)	Post-epidemic group M (P25, P75)	*t*/*Z*	*P*
TT, s	17.0 (15.5, 18.96)	16.1 (14.4, 18.4)	−5.50	<0.001
PT, s	11.4 (10.5, 12.5)	11.8 (10.9, 12.8)	−3.92	<0.001
PT-INR	1.1 (0.97, 1.2)	1.1 (1.0, 1.2)	−3.77	<0.001
APTT, s	29.4 (26, 34.7)	30.4 (26.4, 36.1)	−2.17	0.03
FBG, g/L	3.1 (2.5, 4.0)	3.2 (2.5, 4.1)	−0.32	0.75
D-Dimer, g/ml	1.7 (0.8, 3.4)	1.7 (0.8, 3.4)	−0.37	0.72
WBC, ×10^9^/L	7.0 (5.4, 10.1)	7.5 (5.6, 10.6)	−1.59	0.11
RBC, ×10^9^/L	3.7 (3.0, 4.2)	3.8 (3.1, 4.3)	−1.33	0.19
HGB, g/L	111 (91, 124)	114 (91, 128)	−1.42	0.16
PLT, ×10^9^/L	213 (152, 272)	199 (148, 254)	−1.63	0.10
ALB, g/L	31 (26, 35)	30 (27, 34)	−0.83	0.41
GLO, g/L	28 (25, 32)	28 (25, 32)	−1.28	0.20
ALT, U/L	19 (13, 36)	21 (13, 32)	−0.61	0.54
AST, U/L	22 (16, 38)	23 (16, 34)	−0.03	0.98
LDH, U/L	201 (169, 273)	205 (167, 270)	−0.31	0.76
Urea, mmol/L	5.8 (4.1, 9.8)	6.0 (4.4, 9.5)	−1.32	0.19
CREA, μmol/L	68 (52, 107)	74 (56, 109)	−1.88	0.06
URIC, μmol/L	291 (209, 393)	288 (200, 403)	−0.48	0.63
HbA1c, %	6.1 (5.7, 6.9)	6.4 (5.7, 7.3)	−3.19	0.001

TT, thrombin time; PT, prothrombin time; PT-INR, PT-international normalized ratio; APTT, activated partial thrombin time; FBG, fasting blood glucose; WBC, white blood cell count; RBC, red blood cell count; HGB, hemoglobin (HGB); PLT, platelets, ALB, albumin, GLO, globulin; ALT, alanine aminotransferase; AST, aspartate aminotransferase; LDH, lactate dehydrogenase; CREA, creatinine; URIC, uric acid and HbA1c, glycosylated hemoglobin.

### Comparison of lower extremity venous vascular ultrasound results

According to the lower extremity venous vascular ultrasound results (Table 3), there were 100 cases (25.8%) of lower extremity arteriovenous thrombosis in the epidemic group, including 43 cases (43%) on the left side, 30 cases (30%) on the right side, and 27 cases (27%) on both sides. Thrombus localization analysis showed 19 cases (19.0%) with multi-segmental involvement, 2 cases (2.0%) in the femoral vein, 5 cases (5.0%) in the popliteal vein, and 74 cases (74.0%) localized to the muscular calf veins. In the post-epidemic group, lower extremity arteriovenous thrombosis occurred in 189 cases (32.6%), including 54 cases (28.6%) on the left side, 56 cases (29.6%) on the right side, and 79 cases (41.8%) on both sides. Thrombus distribution patterns also varied, with 27 cases (14.3%) demonstrating multi-segmental involvement, 5 cases (2.6%) in the femoral vein, 5 cases (2.6%) in the popliteal vein, and 152 cases (80.4%) localized to the muscular calf veins. The number of lower extremity arteriovenous thrombosis in the post-epidemic group was significantly larger than that in the epidemic group (*P* = 0.036).

**Table 3:** Lower extremity arteriovenous ultrasound in emergency inpatients in epidemic group and post-epidemic group.

Characteristics		Epidemic group, *n* (%)	Post-epidemic group, *n* (%)	*χ* ^2^	*P*
Thrombosis	Yes	100 (25.8%)	189 (32.6%)	4.42	0.036
	No	288 (74.2%)	390 (67.4%)		
Segment					
≥2 vein segments		19 (19%)	27 (14.3%)		
Femoral vein		2 (2%)	5 (2.65%)		
Popliteal vein		5 (5%)	5 (2.65%)		
Muscle calf vein		74 (74%)	152 (80.4%)		

### Binary logistic regression analysis of related indicators affecting lower limb venous thrombosis

Since the portion of patients suffering from lower extremity arteriovenous thrombosis in the post-epidemic group was significantly higher in post-epidemic group, we further performed a logistic regression analysis to evaluate the independent risk factors of lower extremity arteriovenous thrombosis in post-epidemic group. The factors with statistically significant differences in the univariate analysis, including age, smoking history, drinking history, PT, APTT, PT-INR, and HbA1C, were enrolled in the logistic regression analysis. The results suggested that age, smoking history, drinking history and HbA1C were independent risk factors for thrombosis (*P* < 0.05, Table 4).

**Table 4:** Multivariate logistic regression analysis of risk factors for lower limb venous thrombosis in post-epidemic group.

Characteristics	*B*	*P*	OR	95% CI	
				Lower limit	Upper limit
Age	0.86	<0.001	2.37	1.47	3.82
Smoking history	0.98	<0.001	2.66	1.85	3.81
Drinking history	0.70	<0.001	2.02	1.40	2.90
Sclerosis	−0.41	0.28	0.66	0.32	1.39
TT	0.01	0.65	1.01	0.97	1.05
PT	−0.06	0.05	0.94	0.86	1.03
PT-INR	0.36	0.41	1.43	0.61	3.35
HbA1c	0.10	0.021	1.11	1.02	1.21
Constant	−3.30	<0.001	0.04		

TT, thrombin time; PT, prothrombin time; PT-INR, PT-international normalized ratio; HbA1c, glycosylated hemoglobin; OR, odds ratio and CI, confidence interval.

### Prediction model of lower extremity arteriovenous thrombosis

The prediction model was established as follow:

prellogit(P)=0.863×age+0.978×smokinghistory+0.702×drinkinghistory+0.104×HbA1C−2.439

**Figure 1:**
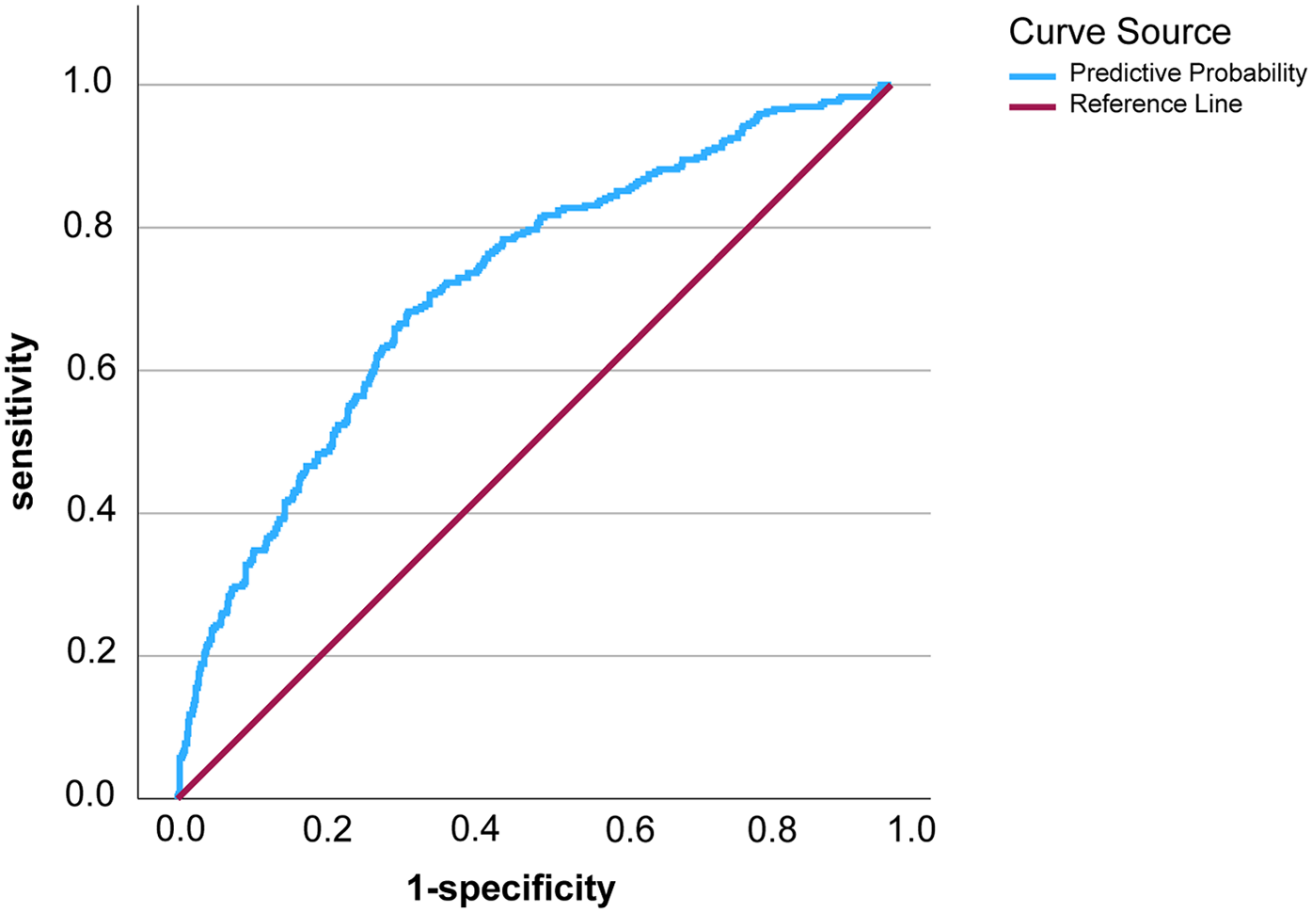
The ROC curve of the prediction model for lower extremity deep vein thrombosis in post-epidemic era.

ROC curve analysis of the predictive model showed that the area under ROC curve (AUC) of the model was 0.718, the cut-off value was 0.271, Jorden index was 0.359, sensitivity was 0.682, and specificity was 0.677 (Figure 1). These results indicated that the model could effectively assess the risk of LEDVT in emergency patients of post-epidemic group. Hosmer-Lemeshow goodness of fit test was further carried out and resulted in a *P* value of 0.055, suggesting the prediction model had a good accuracy.

## Discussion

PASC, or long COVID, have an impact on many organ systems such as respiration, circulation, digestion, endocrinology, urology, and neuropsychiatry (13–15). It is reported that COVID-19 could directly damage endothelial cells, leading to coagulation dysfunction and promoting the occurrence and development of multiple organ failure (16). A higher risk of venous thromboembolism events in patients with COVID-19 infection has been reported (17). Research indicate that the incidence of venous thromboembolism in patients hospitalized for COVID-19 pneumonia in non-ICU settings can be as high as 20% (18–20). In one study conducted in New York, the rate of DVT in COVID-19 hospitalized patients was found to be 31%, compared to 19% in hospitalized patients without COVID-19 (21). However, data regarding the occurrence of venous thromboembolism events, especially the incidence of DVT in emergency inpatients in the post- epidemic era are scant. In view of the poor prognosis and high risk of death of LEDVT, it is particularly important to determine the incidence of LEDVT in the post-epidemic era and establish a prediction model for the risk of LEDVT.

This study showed that the incidence of LEDVT in emergency inpatients in the post-epidemic era was 33.2%, which was significantly higher than that in the epidemic period (26.8%). Meanwhile, compared with the epidemic period, PT, PT-INR, APTT and HbA1c became the high-risk factors for LEDVT in emergency patients in the post-epidemic era. Prior research has identified age as a significant risk factor for thrombotic cardiovascular complications in both arterial (acute myocardial infarction, stroke) and venous (deep vein thrombosis, pulmonary embolism) systems (22). Aging in humans is associated with elevated levels of plasma coagulation proteins or antifibrinolytic factors. As individuals age, the production of proteins C and S declines more rapidly than that of coagulation factors, leading to a hypercoagulable state in the elderly (23). Lifestyle factors, such as smoking and alcohol consumption, have also been identified as independent risk factors for thrombosis. Smoking contributes to thrombosis through multiple mechanisms, including endothelial damage, increased platelet activation, and elevated blood viscosity, while excessive alcohol intake disrupts endothelial function, promotes oxidative stress, and induces metabolic abnormalities, all of which increase thrombosis risk (24–26). As a cornerstone biomarker for diabetes management, elevated HbA1c levels reflect chronic hyperglycemia that fosters a prothrombotic state through inflammatory pathways and impaired fibrinolysis, particularly via upregulation of plasminogen activator inhibitor-1 (PAI-1) (27, 28). Despite these established associations, the precise mechanisms underlying the increased LEDVT incidence in the post-pandemic era warrant further investigation. We speculated that the treatment and prevention strategy of COVID-19 might be the causative factors. During the epidemic period, the screening and treatment strategies of SARS-CoV-2 were very strict in China. None of the patients admitted to the ward were COVID-19 patients. However, during the post-epidemic period, the COVID-19 was not tested and the patients who have been or was being infected with COVID-19 increased significantly. It is well documented that SARS-CoV-2 is characterized by elevated D-dimers and other bio humoral markers and is associated with coagulopathy (29, 30). In this context, the proportion of people infected with COVID-19 in the past or now will increase, and the hypercoagulability caused by COVID-19 itself will increase the incidence of muscle DVT. That's what we thought at first, but there's a hard thing: there's no accurate percentage of infections. In addition, PLT, hemoglobin, PT, APTT, fibrinogen and D-dimer were recommended to be tested in all hospitalized patients with COVID-19. It is recommended that unless contraindicated, all hospitalized patients with COVID-19 should receive thromboprophylaxis such as low molecular weight heparin or regular heparin (31). Anticoagulation during epidemic period not only decrease the mortality and curtailed viral persistence, but also decrease the incidence of DVT to some extent. In the post-epidemic era, the SAR-CoV-2 was not tested and anticoagulation treatment was significantly reduced. This might lead to the increase in LEDVT. It is also reported some mRNA vaccines could lead to thrombosis events (32). Besides, the different SARS-CoV-2 variants on coagulopathy might also be a reason. However, the role of the different SARS-CoV-2 variant or the effects of vaccines on the incidence of DVT is hardly to investigate due to the lack of attention to COVID-19.

In recent years, growing evidence has supported the integration of routine clinical characteristics and laboratory examinations into clinical decision-making for thrombosis risk assessment (33). Over decades, researchers have dedicated efforts to developing predictive models for DVT risk by synthesizing diverse predictors, including demographic data, medical history, and laboratory biomarkers. For instance, by retrospectively analyzing 3381 eligible patients in a primary care setting, Shekarchian et al. developed a risk score incorporating D-dimer levels, Wells score, age, gender, family history of venous thromboembolism, and anticoagulation use, achieving a sensitivity and specificity of 82% in distinguishing DVT among patients with suspected DVT (34). Similarly, Violi et al. proposed a scoring system based on age, D-dimer, and albumin levels, which exhibited good predictive performance for thrombotic events, with an AUC of 0.752 (35). Notably, Zhang et al. developed a nomogram integrating age, mean corpuscular hemoglobin concentration D-dimer, platelet distribution width, and thrombin time to assess postoperative DVT risk following lower extremity orthopedic surgery. The model showed robust discriminative ability, with AUC values of 0.859 and 0.857 in the training and validation cohorts, respectively (36). However, to date, no studies have reported predictive models for the occurrence of LEDVT in the post-pandemic era. In this study, we developed a predictive model incorporating age, smoking history, alcohol consumption history, and HbA1c levels, achieving an AUC of 0.718. This suggests that the model demonstrates good accuracy in predicting the occurrence of LEDVT in the post-pandemic era.

There are some limitations in this study. First, this is a small sample size study conducted in a single center. Second, as a retrospective study, certain data were incomplete, such as the lack of further stratification regarding thrombus location and the potential role of arterial diseases, which warrants additional investigation in future research. Third, the grouping of patients into epidemic group and post-epidemic group was artificial. Forth, the prediction model should be validated in a large-size, independent cohorts in multi-centers.

In conclusion, in the post-pandemic era, we should still be aware of the risk of blood clots in people who are old, have a history of smoking or drinking, have combined diabetes, and have poor blood glucose control. Exact predict the occurrence of LEDVT in emergency inpatients could assist medical professional for timely intervention and reduce the risk of further harm.

## Data Availability

The original contributions presented in the study are included in the article/Supplementary Material, further inquiries can be directed to the corresponding authors.
